# Crystal structure of (*E*)-4,4,4-tri­fluoro-3-phenyl­but-2-enoic acid

**DOI:** 10.1107/S2056989015023725

**Published:** 2015-12-31

**Authors:** Alexey Barkov

**Affiliations:** a Department of Chemistry, Institute of Natural Sciences, Ural Federal University, pr. Lenina 51, 620000 Ekaterinburg, Russian Federation

**Keywords:** crystal structure, tri­fluoro­methyl acid, hydrogen bonding

## Abstract

In the title compound, C_10_H_7_F_3_O_2_, the dihedral angle between the benzene ring and the ethyl­ene plane is 76.34 (11)°. In the crystal, O—H⋯O hydrogen bonds link the mol­ecules into *C*(4) chains propagating in [010].

## Scheme   

The title compound is shown below.
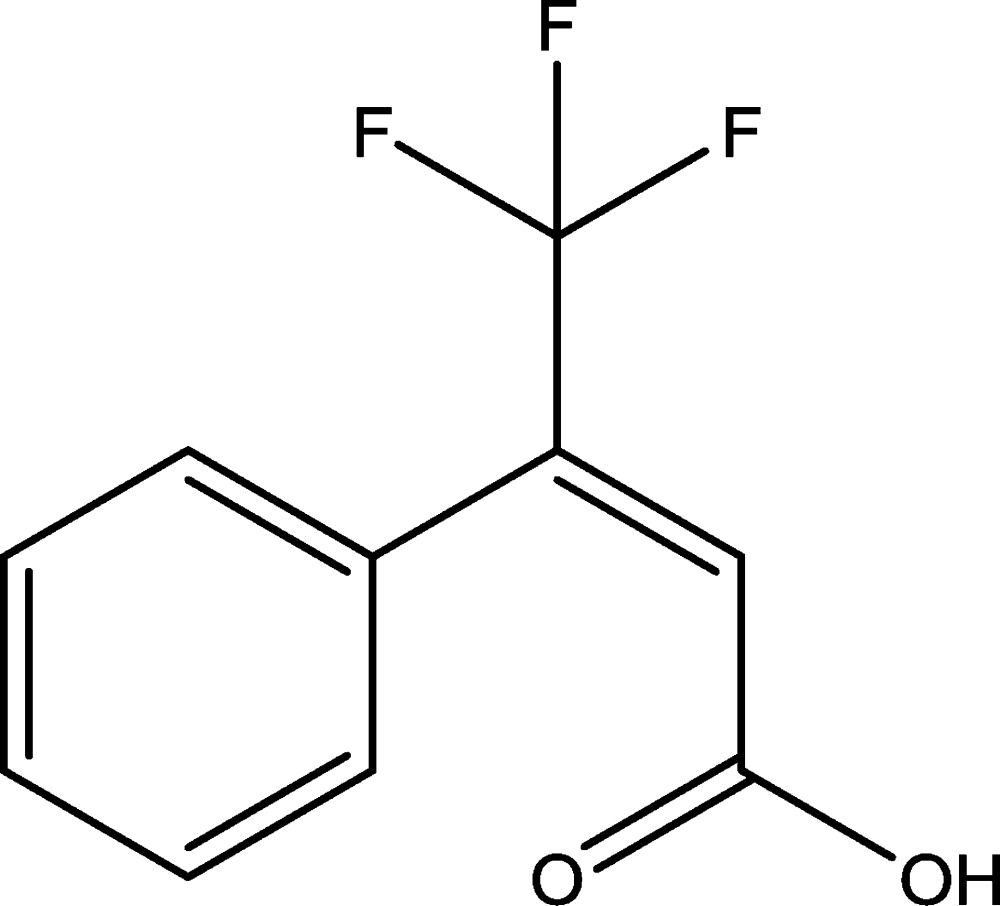


## Experimental   

### Crystal data  

C_10_H_7_F_3_O_2_
*M* *_r_* = 216.16
Monoclinic, 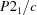

*a* = 11.4093 (9) Å
*b* = 5.7749 (4) Å
*c* = 14.7469 (8) Å
β = 96.300 (6)°
*V* = 965.77 (11) Å^3^
*Z* = 4
Mo *K*α radiation
μ = 0.14 mm^−1^
*T* = 295 K
0.25 × 0.12 × 0.03 mm

### Data collection  

Agilent Xcalibur, Eos diffractometer
Absorption correction: multi-scan (*CrysAlis PRO*; Agilent, 2013 ▸) *T*_min_ = 0.835, *T*_max_ = 1.000
3799 measured reflections
1960 independent reflections
1252 reflections with *I* > 2σ(*I*)
*R*_int_ = 0.022

### Refinement  

*R*[*F* ^2^ > 2σ(*F* ^2^)] = 0.047
*wR*(*F* ^2^) = 0.171
*S* = 1.02
1960 reflections
140 parameters
H atoms treated by a mixture of independent and constrained refinement
Δρ_max_ = 0.17 e Å^−3^
Δρ_min_ = −0.25 e Å^−3^

### 

Data collection: *CrysAlis PRO* (Agilent, 2013 ▸); cell refinement: *CrysAlis PRO*; data reduction: *CrysAlis PRO*; program(s) used to solve structure: *SUPERFLIP* (Palatinus & Chapuis, 2007 ▸); program(s) used to refine structure: *SHELXL97* (Sheldrick, 2008 ▸); molecular graphics: *OLEX2* (Dolomanov *et al.*, 2009 ▸); software used to prepare material for publication: *OLEX2*.

## Supplementary Material

Crystal structure: contains datablock(s) I. DOI: 10.1107/S2056989015023725/hb7527sup1.cif

Structure factors: contains datablock(s) I. DOI: 10.1107/S2056989015023725/hb7527Isup2.hkl

Supporting information file. DOI: 10.1107/S2056989015023725/hb7527Isup3.cdx

Supporting information file. DOI: 10.1107/S2056989015023725/hb7527Isup4.cml

**Fig. 1.**
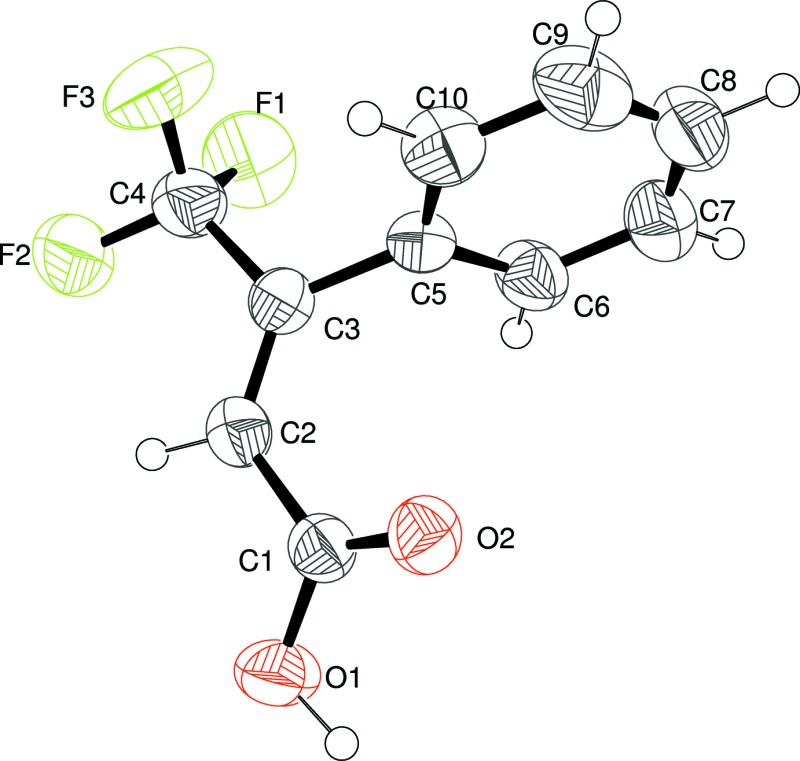
Ellipsoid plot.

. DOI: 10.1107/S2056989015023725/hb7527fig1.tif
Ellipsoid plot.

CCDC reference: 1441578

Additional supporting information:  crystallographic information; 3D view; checkCIF report

## Figures and Tables

**Table 1:** Hydrogen-bond geometry (Å, °)

*D*—H⋯*A*	*D*—H	H⋯*A*	*D*⋯*A*	*D*—H⋯*A*
O1—H1⋯O2^i^	0.97 (3)	1.77 (3)	2.715 (2)	166 (3)

Symmetry code: (i) 
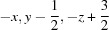
.

## supplementary crystallographic information

### S1. Refinement

The OH H atom was freely refined. C-bound H atoms were positioned geometrically and refined using a riding model with *d*(C—H) = 0.93–0.97 Å and *U*_iso_(H) = 1.2*U*_eq_(C).

### Crystal data

**Table:**

C_10_H_7_F_3_O_2_	*F*(000) = 440
*M**_r_* = 216.16	*D*_x_ = 1.487 Mg m^−^^3^
Monoclinic, *P*2_1_/*c*	Mo *K*α radiation, λ = 0.7107 Å
*a* = 11.4093 (9) Å	Cell parameters from 816 reflections
*b* = 5.7749 (4) Å	θ = 2.8–24.2°
*c* = 14.7469 (8) Å	µ = 0.14 mm^−^^1^
β = 96.300 (6)°	*T* = 295 K
*V* = 965.77 (11) Å^3^	Plate, colourless
*Z* = 4	0.25 × 0.12 × 0.03 mm

### Data collection

**Table:**

Agilent Xcalibur, Eos diffractometer	1960 independent reflections
Radiation source: Enhance (Mo) X-ray Source	1252 reflections with *I* > 2σ(*I*)
Graphite monochromator	*R*_int_ = 0.022
Detector resolution: 15.9555 pixels mm^-1^	θ_max_ = 26.4°, θ_min_ = 1.8°
ω scans	*h* = −14→11
Absorption correction: multi-scan (*CrysAlis PRO*; Agilent, 2013)	*k* = −7→6
*T*_min_ = 0.835, *T*_max_ = 1.000	*l* = −18→18
3799 measured reflections	

### Refinement

**Table:**

Refinement on *F*^2^	Primary atom site location: iterative
Least-squares matrix: full	Secondary atom site location: difference Fourier map
*R*[*F*^2^ > 2σ(*F*^2^)] = 0.047	Hydrogen site location: inferred from neighbouring sites
*wR*(*F*^2^) = 0.171	H atoms treated by a mixture of independent and constrained refinement
*S* = 1.02	*w* = 1/[σ^2^(*F*_o_^2^) + (0.1*P*)^2^] where *P* = (*F*_o_^2^ + 2*F*_c_^2^)/3
1960 reflections	(Δ/σ)_max_ < 0.001
140 parameters	Δρ_max_ = 0.17 e Å^−^^3^
0 restraints	Δρ_min_ = −0.25 e Å^−^^3^

### Special details

**Table:**

Experimental. Absorption correction: CrysAlisPro, Agilent Technologies, Version 1.171.36.32 (release 02-08-2013 CrysAlis171 .NET) (compiled Aug 2 2013,16:46:58) Empirical absorption correction using spherical harmonics, implemented in SCALE3 ABSPACK scaling algorithm.
Geometry. All e.s.d.'s (except the e.s.d. in the dihedral angle between two l.s. planes) are estimated using the full covariance matrix. The cell e.s.d.'s are taken into account individually in the estimation of e.s.d.'s in distances, angles and torsion angles; correlations between e.s.d.'s in cell parameters are only used when they are defined by crystal symmetry. An approximate (isotropic) treatment of cell e.s.d.'s is used for estimating e.s.d.'s involving l.s. planes.
Refinement. Refinement of *F*^2^ against ALL reflections. The weighted *R*-factor *wR* and goodness of fit *S* are based on *F*^2^, conventional *R*-factors *R* are based on *F*, with *F* set to zero for negative *F*^2^. The threshold expression of *F*^2^ > σ(*F*^2^) is used only for calculating *R*-factors(gt) *etc*. and is not relevant to the choice of reflections for refinement. *R*-factors based on *F*^2^ are statistically about twice as large as those based on *F*, and *R*- factors based on ALL data will be even larger.

### Fractional atomic coordinates and isotropic or equivalent isotropic displacement parameters (Å^2^)

**Table:**

	*x*	*y*	*z*	*U*_iso_*/*U*_eq_	
F1	0.33889 (15)	0.9642 (4)	0.53424 (11)	0.0878 (6)	
F2	0.16331 (17)	1.0064 (3)	0.47259 (11)	0.0956 (7)	
F3	0.23597 (19)	1.2390 (3)	0.57582 (12)	0.0938 (6)	
O1	−0.01828 (17)	0.4089 (3)	0.62622 (12)	0.0651 (6)	
H1	−0.042 (3)	0.311 (6)	0.674 (2)	0.101 (11)*	
O2	0.06645 (14)	0.5790 (3)	0.75208 (10)	0.0510 (5)	
C1	0.04817 (19)	0.5707 (4)	0.66953 (15)	0.0439 (6)	
C2	0.0965 (2)	0.7311 (4)	0.60493 (15)	0.0484 (6)	
H2	0.0581	0.7394	0.5460	0.058*	
C3	0.19045 (19)	0.8642 (4)	0.62488 (14)	0.0435 (5)	
C4	0.2304 (2)	1.0167 (5)	0.55131 (17)	0.0565 (7)	
C5	0.26860 (18)	0.8691 (4)	0.71297 (14)	0.0408 (5)	
C6	0.3474 (2)	0.6892 (4)	0.73361 (17)	0.0536 (6)	
H6	0.3493	0.5657	0.6933	0.064*	
C7	0.4226 (2)	0.6914 (5)	0.81282 (19)	0.0683 (8)	
H7	0.4753	0.5699	0.8258	0.082*	
C8	0.4205 (3)	0.8706 (6)	0.87260 (18)	0.0703 (8)	
H8	0.4713	0.8706	0.9264	0.084*	
C9	0.3438 (3)	1.0505 (5)	0.85370 (18)	0.0719 (8)	
H9	0.3428	1.1728	0.8947	0.086*	
C10	0.2672 (2)	1.0519 (4)	0.77358 (17)	0.0555 (7)	
H10	0.2153	1.1748	0.7608	0.067*	

### Atomic displacement parameters (Å^2^)

**Table:**

	*U*^11^	*U*^22^	*U*^33^	*U*^12^	*U*^13^	*U*^23^
F1	0.0745 (12)	0.1200 (16)	0.0741 (11)	−0.0052 (10)	0.0318 (9)	0.0103 (10)
F2	0.1015 (14)	0.1180 (16)	0.0611 (10)	−0.0450 (11)	−0.0194 (9)	0.0354 (10)
F3	0.1397 (17)	0.0535 (10)	0.0926 (13)	−0.0185 (10)	0.0318 (11)	0.0081 (10)
O1	0.0751 (13)	0.0738 (13)	0.0452 (9)	−0.0342 (10)	0.0011 (8)	0.0002 (9)
O2	0.0586 (11)	0.0528 (10)	0.0413 (9)	−0.0007 (7)	0.0039 (7)	−0.0017 (7)
C1	0.0409 (13)	0.0449 (13)	0.0454 (12)	0.0004 (9)	0.0022 (9)	−0.0006 (10)
C2	0.0481 (14)	0.0533 (14)	0.0424 (11)	−0.0035 (11)	−0.0016 (9)	0.0031 (11)
C3	0.0436 (13)	0.0414 (12)	0.0452 (12)	0.0013 (10)	0.0039 (9)	0.0006 (10)
C4	0.0573 (16)	0.0594 (16)	0.0526 (14)	−0.0103 (12)	0.0050 (11)	0.0034 (12)
C5	0.0394 (12)	0.0399 (12)	0.0435 (11)	−0.0034 (9)	0.0068 (9)	−0.0021 (10)
C6	0.0513 (14)	0.0470 (14)	0.0605 (14)	0.0025 (11)	−0.0030 (11)	−0.0087 (12)
C7	0.0594 (17)	0.0656 (18)	0.0750 (18)	0.0028 (13)	−0.0138 (13)	0.0047 (16)
C8	0.0666 (19)	0.087 (2)	0.0536 (15)	−0.0150 (17)	−0.0080 (13)	0.0038 (16)
C9	0.084 (2)	0.077 (2)	0.0546 (15)	−0.0144 (17)	0.0094 (14)	−0.0261 (15)
C10	0.0611 (16)	0.0498 (14)	0.0566 (14)	0.0052 (12)	0.0114 (11)	−0.0091 (12)

### Geometric parameters (Å, º)

**Table:**

F1—C4	1.326 (3)	C5—C6	1.386 (3)
F2—C4	1.320 (3)	C5—C10	1.385 (3)
F3—C4	1.333 (3)	C6—H6	0.9300
O1—H1	0.96 (3)	C6—C7	1.371 (3)
O1—C1	1.323 (3)	C7—H7	0.9300
O2—C1	1.213 (2)	C7—C8	1.361 (4)
C1—C2	1.478 (3)	C8—H8	0.9300
C2—H2	0.9300	C8—C9	1.367 (4)
C2—C3	1.326 (3)	C9—H9	0.9300
C3—C4	1.506 (3)	C9—C10	1.390 (4)
C3—C5	1.493 (3)	C10—H10	0.9300
			
C1—O1—H1	105.0 (18)	C10—C5—C3	121.9 (2)
O1—C1—C2	111.47 (19)	C10—C5—C6	118.9 (2)
O2—C1—O1	122.6 (2)	C5—C6—H6	119.7
O2—C1—C2	125.9 (2)	C7—C6—C5	120.7 (2)
C1—C2—H2	117.6	C7—C6—H6	119.7
C3—C2—C1	124.7 (2)	C6—C7—H7	119.8
C3—C2—H2	117.6	C8—C7—C6	120.3 (3)
C2—C3—C4	118.7 (2)	C8—C7—H7	119.8
C2—C3—C5	126.5 (2)	C7—C8—H8	119.9
C5—C3—C4	114.63 (19)	C7—C8—C9	120.1 (3)
F1—C4—F3	104.7 (2)	C9—C8—H8	119.9
F1—C4—C3	111.4 (2)	C8—C9—H9	119.8
F2—C4—F1	106.6 (2)	C8—C9—C10	120.4 (2)
F2—C4—F3	106.7 (2)	C10—C9—H9	119.8
F2—C4—C3	114.5 (2)	C5—C10—C9	119.6 (2)
F3—C4—C3	112.2 (2)	C5—C10—H10	120.2
C6—C5—C3	119.22 (19)	C9—C10—H10	120.2

### Hydrogen-bond geometry (Å, º)

**Table:**

*D*—H···*A*	*D*—H	H···*A*	*D*···*A*	*D*—H···*A*
O1—H1···O2^i^	0.97 (3)	1.77 (3)	2.715 (2)	166 (3)

Symmetry code: (i) −*x*, *y*−1/2, −*z*+3/2.
