# Supplementary figures and images for: Crystal structure of (E)-4,4,4-tri­fluoro-3-phenyl­but-2-enoic acid
Source: Acta Crystallogr E Crystallogr Commun. 2015 Dec 31;71(Pt 12):o1090. doi: 10.1107/S2056989015023725 (PMC4719994; doi:10.1107/S2056989015023725)

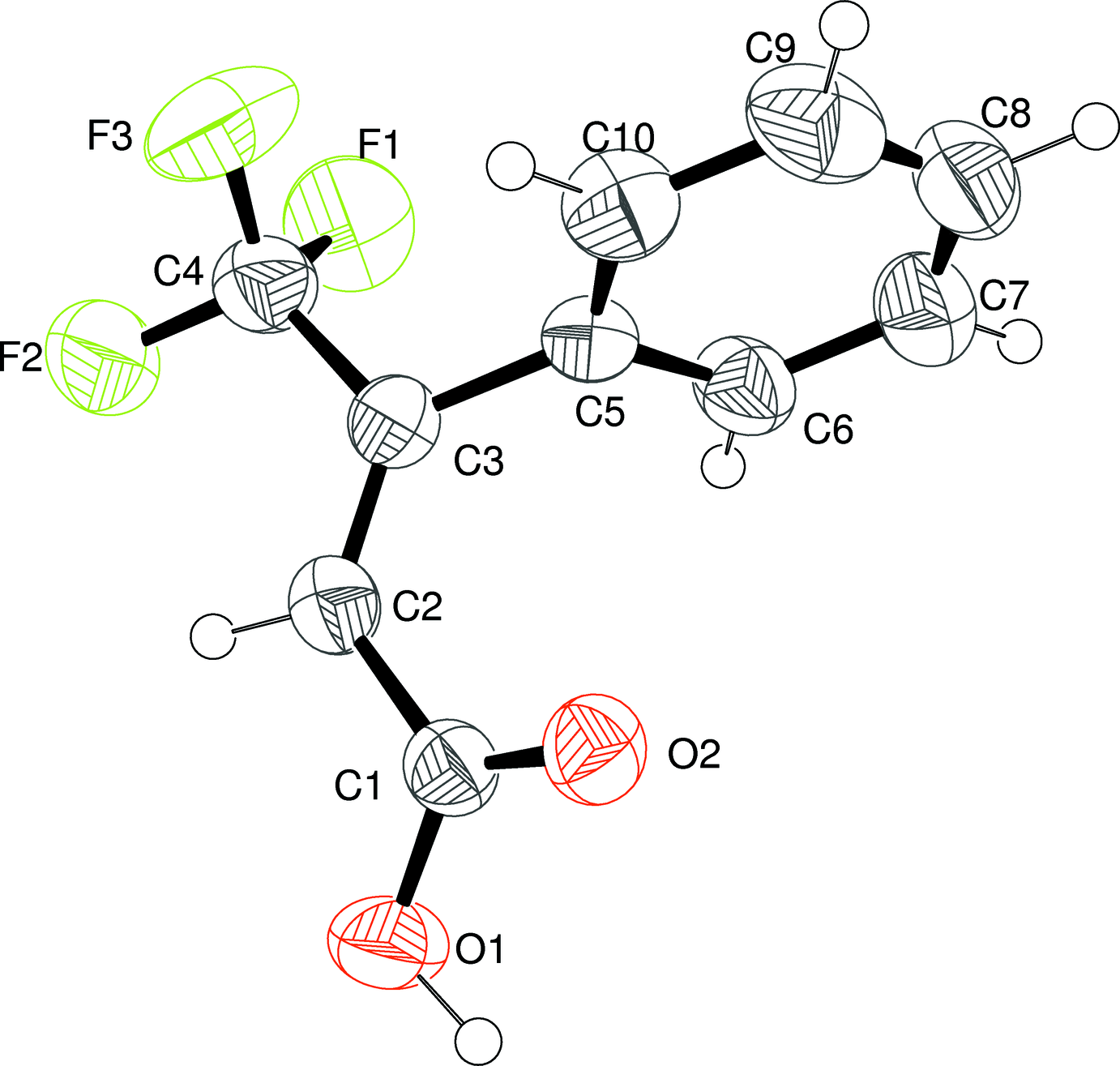

Supplement: Supplementary file 5 [file e-71-o1090-fig1.tif]
